# Electrical Model of a Carbon-Polymer Composite (CPC) Collision Detector

**DOI:** 10.3390/s120201950

**Published:** 2012-02-10

**Authors:** Karl Kruusamäe, Andres Punning, Alvo Aabloo

**Affiliations:** IMS Lab, Institute of Technology, University of Tartu, Nooruse 1, 50411 Tartu, Estonia; E-Mails: andres.punning@ut.ee (A.P.); alvo.aabloo@ut.ee (A.A.)

**Keywords:** carbon, CDC, polymer, ionic liquid, self-sensing, actuator, sensor, smart material

## Abstract

We present a study of an electrical model of electromechanically active carbon-polymer composite (CPC) with carbide-derived carbon (CDC) electrodes. The major focus is on investigation of surface electrode behavior upon external bending of the material. We show that electrical impedance measured from the surface of the CDC-based CPC can be used to determine the curvature of the material and, hence, the tip displacement of a CPC laminate in a cantilever configuration. It is also shown that by measuring surface signals in the process of an actuator’s work-cycle, we obtain a self-sensing collision-detecting CPC actuator that can be considered as a counterpart of biomimetic vibrissae.

## 1. Introduction

Electromechanically active polymers (EAP) alter their shape or size when an electric stimulus is applied. Ionic EAP (IEAP) is one of two main classes of electroactive materials, where actuation is primarily caused by the displacement of ions inside a polymer matrix [1]. Perhaps the best known ionic EAP is the ionic polymer-metal composite (IPMC) [2,3].

In Nature, vibrissae (also known as whiskers) are sensory hairs that respond to mechanical stimuli such as displacement, tension and pressure [4,5]. Vibrissae serve as tactile sensors for mammals such as domestic cats, harbor seals, *etc*. by helping navigation in the dark. Moreover, even object recognition is proposed as one of the functions and capability of vibrissae arrays [5]. This paper proposes the creation of biomimetic vibrissae as one possible application area for electromechanically active materials.

In recent years the range of ionic EAP materials has been extended significantly by means of materials used for electrodes, separator membrane, as well as the electrolyte [6–12]. At first glance, all ionic EAPs seem similar in construction—two conducting electrodes separated by a polymer membrane, containing freely moving ions—but their actuation mechanisms can be significantly different.

Lately, carbon and its many allotropes (e.g., carbon nanotubes, fullerenes, graphene, carbon aerogels, nanoporous carbon, *etc*.) have become very promising materials for electrodes in several electroactive devices, including IEAP. One of the concepts behind the contribution of porous carbon to the electroactivity of a material is related to the electric double layer emerging at the boundary between carbon and electrolyte, therefore the surface area of this boundary is of great importance. Due to its high specific surface area, nanoporous carbon is one of the most widely used materials for electrodes in electric double-layer capacitors (EDLC) [13].

**Figure 1.**
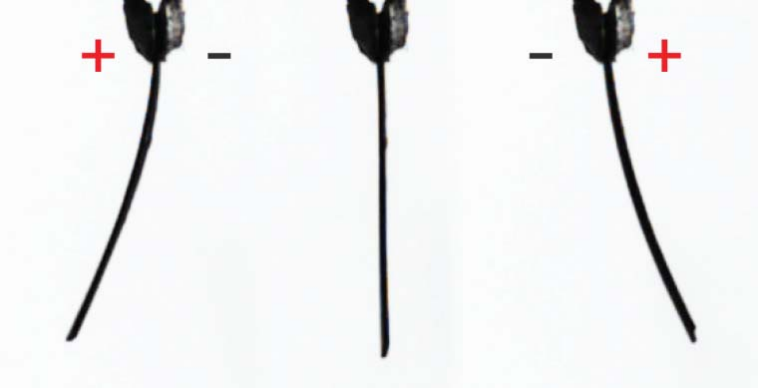
CPC under electric stimulus, *i.e.*, the behavior of the CPC actuator.

Carbon-polymer composites (CPCs) are a type of ionic electromechanically active structure, similar to EDLCs in the sense of assembly, as well as the working principle. They consist of two porous carbon electrodes separated by an ion-permeable polymer film. The whole CPC structure—separator membrane and electrodes—contains an ionic liquid as electrolyte. Due to a combination of different charging and ionic effects when relatively low voltage is applied between the surface electrodes, the CPC laminate bends, *i.e.*, it behaves as an actuator (Figure 1). A 3-by-1 cm strip of CPC actuator can exhibit a blocking force of up to 80 mN and achieve its peak tip displacement (over 1 cm) in 15 to 90 seconds.

Nearly all ionic EAPs also function as sensors: they generate a voltage or electric current when stimulated mechanically [14]. Despite the fact that CPC materials are no exception to this rule [15], thus far, a relatively small amount of research regarding the sensorial properties of carbon-based actuators has been published.

Furthermore, electromechanically active materials (including CPCs) are considered attractive for biomimetic applications as they are soft, noiseless and can be cut into various sizes and shapes [16]. Additionally, some of the key advantages of CPC actuators include low operating voltage and the absence of metal parts.

However, in order to utilize CPC (and other smart material) actuators in real-life application, one must find a suitable technology for closed-loop control of the actuator. To date, the most typically implemented feedback sources for ionic EAPs are laser displacement sensors [17], video cameras [18], load cells [7], *etc*. Using such feedback devices can, due to their size, cost and complexity, remarkably limit the practical application areas of EAP actuators. It is thus desirable to avoid unwieldy off-board sensors for closed-loop control.

It is also possible to create a bending actuator system where either some type of material parameter or a sophisticated lightweight sensor is used as the feedback upon tip displacement. For example, Leang *et al.* mounted a strain gage on an IPMC surface electrode to track the movement of the actuator [19], while our earlier studies presented a system where a strain-gage-like structure is patterned directly on the surface of an IPMC laminate [20,21]. The latter type of systems are called self-sensing and the concept involves using the same strip of material simultaneously as an actuator and sensor [20,22,23], provided the sensor signal is used for closed-loop control of the actuator.

Considering IPMC, the change in resistance of its surface electrodes due to the actuator’s curvature has been of essence when creating a self-sensing device. Thus, investigating the carbon-based electrode of a CPC would be vastly informative in regards to comparing IPMC and CPC. As mentioned above, CPCs are also very similar to EDLCs and therefore the super capacitive effect cannot be neglected [24]. Based on these similarities we hypothesized that if, in the process of bending, the resistance of the surface alters, so should the capacitance.

In this paper, by means of electrical impedance spectroscopy (EIS), a relation between the shape of a CPC actuator/sensor and its electrical parameters (resistance, impedance, and capacitance) is described. A study into how different components of a CPC actuator affect its behavior and electrical properties is carried out and presented. More specifically, the main focus is placed on investigating these characteristics by measuring signals from the surface electrode of the CPC laminate. Distinctive resistive and capacitive effects emerge on bending of the actuator, thus providing a concept of self-sensing CPC actuator, able to detect obstacles in its path and, hence, act as artificial vibrissae. This paper also extends the concept of self-sensing IPMC to a CPC actuator.

When interpreting the gathered EIS data two ideologically different approaches are used. First, CPC is considered a black-box device and therefore, we only describe different curvature dependent electrical effects that allow utilization of CPC as a sensor. The second approach applies the theory of impedance spectroscopy in order to give detailed electrical model of CPC.

A CPC actuator is a quite complex system composed of an ionic liquid and special carbon derivative in a somewhat sophisticated structure. Both conductive and isolative components are integrated, while electron conductance is present only in carbon layers; on the other hand, ions can move throughout the CPC laminate. For these considerations, in order to describe an accurate electrical model of a CPC laminate, it is also necessary to investigate the properties of the components that make up the CPC actuator. As the current study focuses on carbon electrode behavior and signals measured from it, the electrode material itself is thoroughly investigated.

The remainder of the paper is organized as follows: in Section 2 we briefly describe the CPC material and its fabrication process. In Sections 3 and 4, a series of electrical impedance spectroscopy measurements are presented, resulting in a presumptive equivalent circuit of a CPC actuator and sensor. Section 5 describes a possible application of a CPC actuator-sensor—a groping collision detector.

## 2. Carbon-Polymer Composite

**Figure 2.**
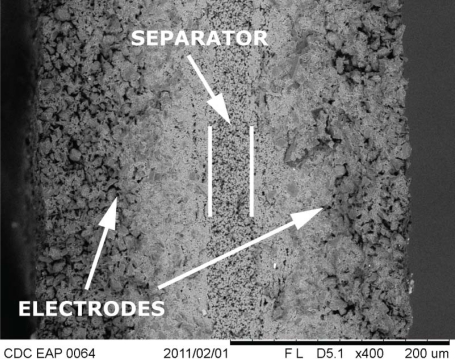
SEM (at 5 kV) image of the cross-section of the CPC laminate.

Carbon-polymer composite (CPC) consists of two porous carbon electrodes separated by an ion-permeable polymer film (Figure 2). The carbon in the electrodes can be carbide-derived carbon (CDC), carbon aerogel, or some other carbon-based material with sufficiently large specific surface area. The role of the separator is to act as a reservoir for the electrolyte and to avoid electronic conductance between electrodes [9]. The whole CPC structure—separator membrane and electrodes—contains an ionic liquid as electrolyte. If voltage, in the range of the electrochemical window of the electrolyte, is applied between the surface electrodes, different dilatometric effects emerge on the anode and cathode. As a result the material bends, *i.e.*, it behaves as an actuator (Figure 1).

The general understanding about the working mechanism of a CPC actuator is not conclusive and is the subject of some dispute. As one plausible explanation, an electro-osmotic effect caused by the voltage applied between the carbon electrodes is proposed. In that scenario, the applied voltage causes a migration of cations towards one surface of the material, and anions move in the opposite direction. Due to the difference in dimensions of anions and cations, the unbalanced distribution of ions causes the side with larger ions to stretch, while the opposite surface is compressed [9,17]. This is the process that is registered as a bending of the material. Alternatively, the bending can be initiated by repulsion or expansion of carbon particles in the electrode layer due to different charging effects, such as electron injection and/or double-layer charging [8,9].

The measurements described in the current paper were conducted using a CPC material fabricated in-house. It is composed of boron carbide-derived carbon with a specific surface area of 1,800 m^2^/g, poly(vinylidene fluoride-co-hexafluoropropene)—PVdF(HFP)—as binding polymer and separator, and 1-ethyl-3-methylimidazolium tetrafluoroborate (EMIBF_4_) as the ionic liquid. A more detailed description of the preparation process of such CPC actuators is given by Torop *et al.* [9].

All of the experiments described in this paper were conducted on CPC samples with 10 × 30 mm dimensions. The 150 μm thick CDC electrodes are isolated by a separator with a thickness of 30 μm (Figure 2).

## 3. Correspondence between the Impedance and the Curvature of a CPC Laminate

While the structure and chemical composition of the CPC material is defined by the fabrication process, it is not conclusively clear how these components interact with each other in the sense of electrical signals. In order to obtain a thorough understanding of the internal mechanisms of a CPC, electrical impedance spectroscopy (EIS) is utilized. EIS allows characterization of CPC by an equivalent circuit made up of resistors, capacitors, and possibly inductances. This section implements EIS to the surface electrode of the CPC laminate for the purpose of determining the electrical model of a CPC that can explain some of the processes occurring on the surface electrode in the course of bending.

**Figure 3.**
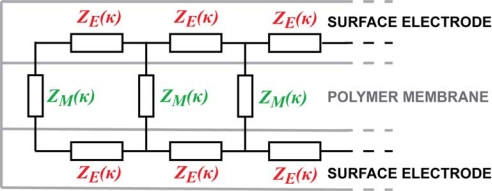
General equivalent circuit of ion-conducting EAP.

Contrarily to the popular lumped models of the EAP devices [25–27], we are looking for the equivalent circuit of the CPC in the form of a transmission line, similar to that of IPMC presented by Punning *et al.* [28]. It consists of an infinite series of similar infinitesimally short equivalent circuits with discrete elements as depicted in Figure 3.

Initially we represent the impedances of the electrodes and the membrane as the general impedances *Z_E_* and *Z_M_*, and expect that some of them show definite correspondence between its value and the curvature of the material. The experiments described hereafter study the whole device as well as the components separately using the electrical impedance spectroscopy (EIS) equipment, while different degrees of bending were imposed to the strip of the material, summarizing with a reasonable equivalent circuit.

### 3.1. Experimental

**Figure 4.**
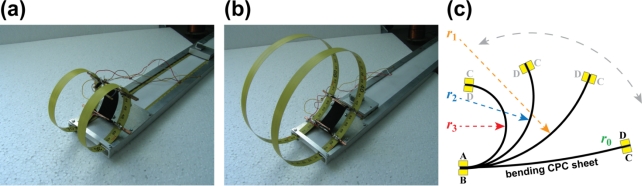
Experimental setup for measuring electrical impedance in respect to the curvature of a CPC.

**Figure 5.**
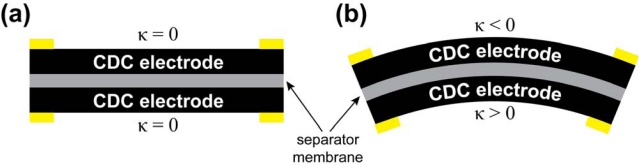
(**a**) Initial state of a CPC laminate; (**b**) Bending of a CPC laminate.

The curvature, *κ* = 1/*r*, of bent CPC is directly related to the displacement of one (*i.e.*, moving) tip of the actuator, since in the cantilever configuration, the other end of the material always remains fixed. The desired curvature was externally imposed to the CPC actuator by means of a special rig that allowed varying the curvature of the CPC laminate in the range of 15 to 100 m^−1^ (Figure 4). Figure 4(a,b) depicts a CPC sample in this customized rig which is enforcing different degrees of curvature. The system allows application of consecutive curvatures to the CPC sample, however, measurements of straight samples cannot be carried out in this type of setup. Figure 5 presents the mechanics of a CPC laminate: while the material is bent, the bottom electrode is notably compressed and the top one expands (Figure 5(b)). In order to measure the effects due to compression and extension of the same surface, the sample needed to be taken out of the system and turned around.

In the course of experiments the CPC was fixed to the rig with four terminals, thus allowing measurements of all necessary signals between the two surfaces as well as along the surface electrodes. While different curvatures were enforced to the CPC sheet, the cross-electrode impedance was measured between terminals A and B (Figure 4(c)), and the corresponding measurements for surface electrodes were conducted between terminals A and D, or B and C respectively. Terminals B and C represent the signals registered from the surface that undergoes expansion and this convexity of an electrode is expressed as the negative curvature. The compression of surface is identifiable as the positive curvature (Figure 5).

A PARSTAT 2273 potentiostat from Princeton Applied Research was used for EIS measurements at a voltage level of 40 mV in the frequency range of 10 mHz–100 kHz. The low voltage that was selected will not cause bending of the actuator, nor does it generate any faradic processes that could deteriorate the integrity of ionic liquid.

### 3.2. Results and Discussion

**Figure 6.**
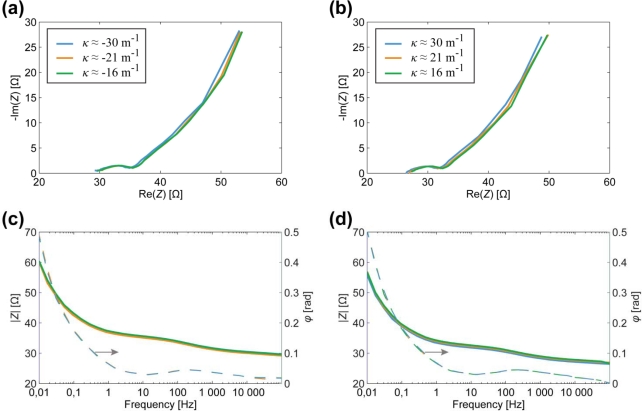
The correlation between the cross-electrode impedance and the curvature of a CPC actuator. (**a**) Nyquist plot for bending in one direction; (**b**) Nyquist plot for bending in another direction; (**c**) Bode plot for bending in one direction; (**d**) Bode plot for bending in another direction.

In Figure 6, the cross-electrode impedance of CPC actuator is depicted. The slightly flattened semicircle observed in Figure 6(a,b) is typically interpreted as a parallel combination of capacitive and resistive components. The steep rise in the imaginary part of *Z* indicates a strong capacitive response of the system. These results comply well to the measurements conducted on another type of CPC device—a bucky-gel SWCNT actuator [24].

As seen in Figure 6, alteration of impedance, depending on the instantaneous curvature, remains within the measurements’ uncertainty of the device. Hence we conclude that this configuration of the CPC cannot be exploited as a sensory or self-sensing device. Next, we try to find the effect from the electrodes in the direction of their length.

**Figure 7.**
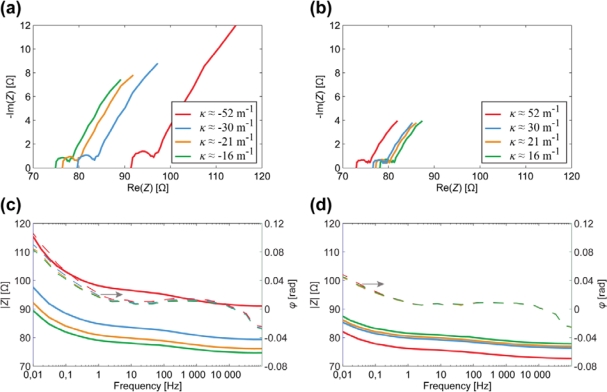
The correlation between the surface electrode impedance and the curvature of a CPC actuator. (**a**) Nyquist plot for convex surface electrode; (**b**) Nyquist plot for concave surface electrode; (**c**) Bode plot for convex surface electrode; (**d**) Bode plot for concave surface electrode.

The results of EIS measurements, conducted on one surface electrode of the whole CPC laminate are depicted in Figure 7. The visual appearance of the curves is pretty similar to those depicted in Figure 6, thus again exhibiting parallel capacitive and resistive components (high frequencies) in series with high capacitance (low frequencies). However, the Nyquist plots given in Figure 7(a,b) show that the curves are scaled along both axes as the curvature of the sample changes. Consequently, both the resistive as well as the capacitive components alter in the course of bending.

**Figure 8.**
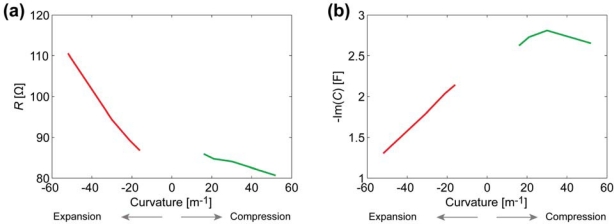
(**a**) The correlation between the surface resistance and the curvature of a CPC at the frequency of 0.2 Hz; (**b**) The correlation between the apparent surface capacitance and the curvature of a CPC at the frequency of 0.2 Hz.

Figure 8(a) depicts the relation between the curvature and the surface electrode resistance of a typical CPC actuator. Similarly to IPMCs [29], when a carbon electrode is stretched, its resistance increases noticeably, while the decrease in resistance of the compressed electrode is relatively small.

After finding the apparent capacitance from the impedance data presented in Figure 7, we see that this empirical capacitance is also dependent on the degree of curvature when measuring surface signals (Figure 8(b)). Even without conclusively understanding the exact electrical model of the CPC material, we can still describe a black-box CPC sensor. As one surface of the CPC actuator is extended, apparent capacitance measured from this surface decreases notably. When the surface is compressed due to the bending of the actuator, the capacitance measured remains nearly unchanged (Figure 8(b)).

**Figure 9.**
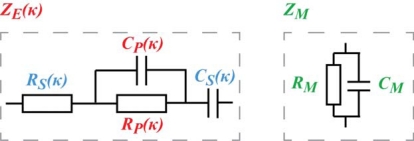
Equivalent circuits for *Z_E_* and *Z_M_*.

The EIS measurements of the cross-electrode impedance and the surface electrode described above cannot lead to a unique definition of the impedances *Z_E_* and *Z_M_* depicted in Figure 3. In the case of a distributed circuit, the appearing capacitance can lie in the electrode as well as in the membrane. The appearing altering impedances described above can be explained in many different possible combinations of curvature-dependent capacitive and resistive components of *Z_E_* and *Z_M_*. One sophisticated combination, proven by electronic circuit simulation software, is obtained when *Z_E_* and *Z_M_* given in Figure 9 are inserted into Figure 3.

## 4. EIS of Individual Components of CPC and a Straightforward Electrical Model

In order to obtain an adequate electrical model of a CPC actuator, it is beneficial to explore each component individually, *i.e.*, to separately test the validity of *Z_E_* and *Z_M_* given in Figure 9. This technique cannot be used in the case of IPMC with chemically deposited electrodes. However, since the composite material investigated in current paper is fabricated in-house and the layers of CPC are created separately and assembled by means of mechanical pressure, it is possible to study the components—membrane and electrode—independently.

The PVdF in a dry form is a good insulator but as the separator membrane for CPC, it is swollen by the ionic liquid. Therefore it is justified to assume that the conductance of the membrane is pure ionic conductance. In that case the equivalent circuit of the membrane—*Z_M_*—consists of only capacitance. Our impedance data on 30–50 μm thick PVdF membrane containing IL, shows only capacitive effect with impedance modulus in the range of dozens of kilo-ohms to mega-ohms, depending on the frequency.

### 4.1. Experimental

**Figure 10.**
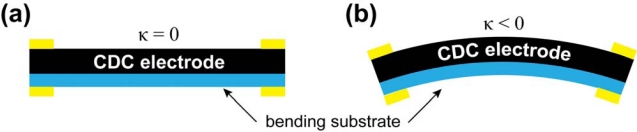
(**a**) Initial state of a CDC-based electrode; (**b**) Bending of a CDC-based electrode.

In order to characterize the correlation between the impedance and curvature of a CDC electrode, the electrode was bonded to a thin poly-carbonate film (Figure 10). In this configuration the impedances of the membrane and the opposite electrode will not interfere with the measurements. Connecting the electrode to a lengthwise inelastic plastic ensures that in the course of external bending the electrode behaves like a regular CPC. That is, the electrode gets compressed when *κ* > 0 and stretched when *κ* < 0, and in comparison to signals measured from the surface of a CPC laminate (Figure 7), any phenomenological difference in results can, thus, be contributed to the opposite electrode and/or separator membrane. The experimental setup was similar to the previous experiments described in Section 3.1.

### 4.2. Results and Discussion

**Figure 11.**
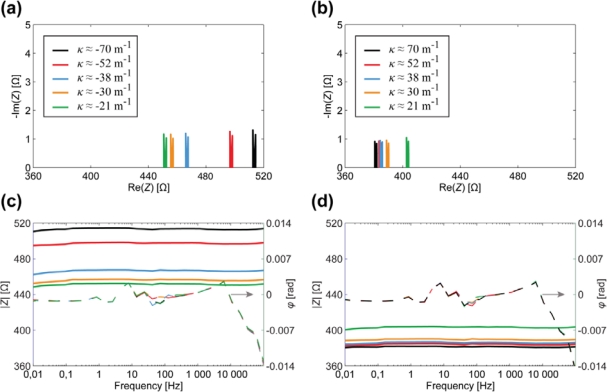
The correlation between the impedance and the curvature of a CDC-based surface electrode. (**a**) Nyquist plot for convex surface electrode; (**b**) Nyquist plot for concave surface electrode; (**c**) Bode plot for convex surface electrode; (**d**) Bode plot for concave surface electrode.

Figure 11 depicts the EIS data registered from the CDC-based electrode (containing ionic liquid and polymer) in case of different degrees of curvature.

**Figure 12.**
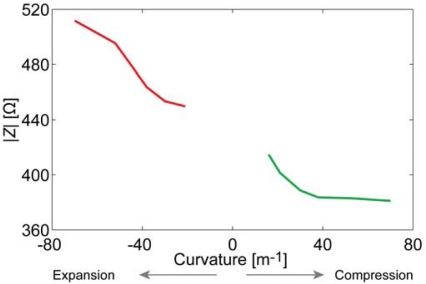
The correlation between the curvature and the impedance modulus of a CDC-based electrode.

The results show that there is no considerable capacitance present in the electrode and demonstrate a clear correlation between the magnitude of impedance and the curvature of a bent electrode. The resistance of a stretching surface increases considerably while the resistance of compressing surface lessens only slightly, as depicted in Figure 12. The change of resistance can be explained by means of the touching surfaces of CDC particles: the concentration of CDC particles in electrode is high enough to ensure pure electronic conductivity. When the already compact electrode gets more compressed, the touching surface of CDC particles increases a little, resulting in the rise of conductivity. When electrode is stretched, some carbon particles are disconnected from each other, leading to decrease in conductivity.

**Figure 13.**
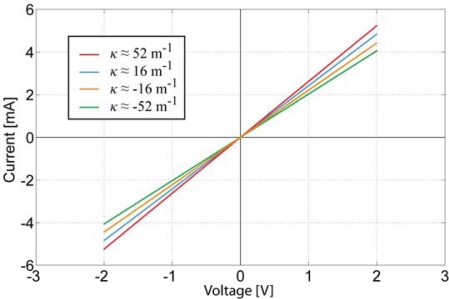
Voltammogram of a CDC-based electrode for different material curvatures.

No relevant capacitive or inductive effect is detected from the CDC-based electrode. The absence of capacitance becomes clearer from the cyclic voltammetry of the same sample given in Figure 13. Despite implementing a wide range of sweep rates (50 mV/s–2 V/s), the voltammogram remained linear, and the only parameter affecting its slope is the curvature of the electrode. This indicates that the concentration of CDC particles in electrode is high enough to not cause any cavities in the conducting surface, even when stretched.

**Figure 14.**
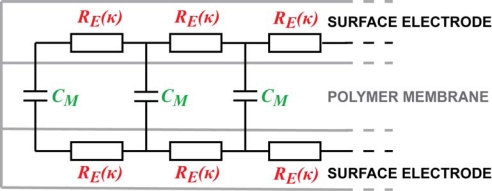
A distributed model of a CPC laminate.

The experiments described in Section 3 showed the presence of apparent capacitive elements in the membrane as well as in the electrodes. Now, based on above described results, we can concretize the components and eliminate capacitance from the electrode. The resulting distributed equivalent circuit of CPC is depicted in Figure 14. The impedance of the electrodes *Z_E_* stands for pure resistivity, while the impedance of the membrane *Z_M_* consists of only a capacitive component. This leads to the understanding that the apparent capacitance in Figures 7 and 8 is registered only due to the coupling effect of the separator membrane. Despite this deduction, the described capacitive phenomenon can still be implemented when using CPC as a black-box position sensor.

Experiments described in Sections 3 and 4 showed that change in resistance of a CDC-based electrode is present both in whole CPC actuator and the electrode alone. However, when measuring surface signals of the actuator, capacitive coupling between surfaces is well registered and, thus, cannot be neglected when creating application utilizing sensorial effects on surface electrode.

The resulting equivalent circuit provide model for a CPC position sensor, self-sensing actuator, or other devices. In current paper we describe self-sensing collision-detecting CPC actuator that can be utilized as biomimetic vibrissae.

## 5. Self-Sensing CPC Actuator Capable of Collision Detection

As stated above, the concept of self-sensitivity involves using the same strip of material simultaneously as an actuator and a sensor, so that sensory signal is used as a feedback for closed-loop control of the actuator. In this way, no external feedback source is required and no significant cost or complexity is added to the system. This section presents a self-sensing CPC collision detector. The device is capable of brandishing the actuator and sensing if it is touching an obstruction. The sensing signal is registered from both surface electrodes of the actuator. Such self-sensing system mimics the functionality of an effective biological tactile sensor—vibrissae.

While Sections 3 and 4 give good characterization of CPC actuator, mere description of sensorial properties of a CPC alone is only the first step towards creation of a self-sensing actuator. Here the challenge lies in measuring all or some of the described phenomena in an electrically active sample.

### 5.1. Experimental

**Figure 15.**
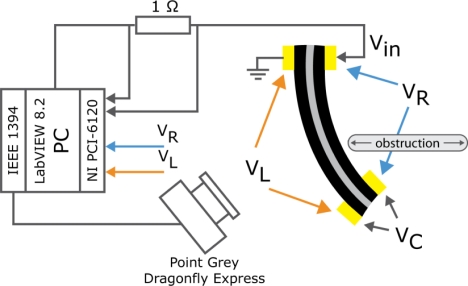
Experimental setup for measuring signals during the work-cycle of a CPC actuator.

For the measurements described in current section, an experimental setup comprising a PC with LabVIEW 8.2 and NI PCI-6120 DAQ card was adopted (Figure 15).

In these measurements a CPC sheet was placed in a cantilever configuration, as depicted in Figure 15. In order to register voltage drops along the surface electrodes, a lightweight clamp with gold contacts was attached to the moving tip of the actuator. The weight of the clamp (less than 100 mg) and the hovering 50-μm-thick wires cause no relevant suppression to the performance of the actuator, capable to lift several grams. A rectangular driving signal (pulse width of 30 s and amplitude of 3 V) was applied to the CPC actuator. The amplitude of the input voltage was chosen to be within the electrochemical window of the ionic liquid, so no disruptive faradic processes take place between the electrodes, nor inside a single electrode. The actuator driving signal *V_in_*, and the voltage drops along the electrodes—*V_L_* and *V_R_*—are measured using the PCI-6120 DAQ board, while the voltage on separator *V_C_* is obtained from the measured results using the arithmetic operations. The electric current was measured as a voltage drop over a series resistor of suitable value, usually about 1 Ω. The curvature and the tip displacement of the actuator were recorded by a Point Grey Dragonfly Express camera. Data acquisition frequency was set to 4 S/s while driving signals (*V_in_*) were generated at the sampling rate of 1 kS/s.

**Figure 16.**
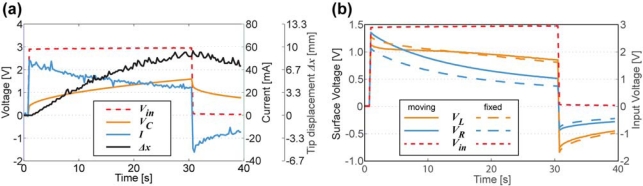
(**a**) Actuator driving signal *V_in_*, voltage drop on separator *V_C_*, current *I*, and tip displacement along the horizontal axis *Δx*; (**b**) Voltage drops along the electrodes of the CPC actuator in unobstructed and fixed configurations.

In the first series of experiments, the strain of CPC sample was caused by the input driving signal. Continuous movement of the tip occurred for the whole 30 seconds of applied voltage, reaching the displacement of ∼10 mm in the direction of horizontal axis (denoted as *Δx* in Figure 16(a)) and ∼2 mm along the vertical axis. Another series of experiments were conducted similarly to the previous one but the CPC sample was mechanically fixed to its initial position. This way, despite the applied voltage, the actuator was unable to bend, thus lessening the effects inherent to the alteration of surface resistance due to the instantaneous curvature of the material. Lastly, experiments where movement of the CPC was obstructed by an entering obstacle at a random moment during the actuation were also carried out.

### 5.2. Results and Discussion

The signals measured simultaneously between different regions of CPC in its electrically active state, *i.e.*, during a single work-cycle of CPC actuator, are depicted in Figure 16. The exponential decay of electric current in Figure 16(a) implies that the separator between two electrodes can be regarded as capacitance with no or negligible shunting conductivity.

The comparison of voltages along the opposite electrodes of both—unobstructed and fixed actuator—are given in Figure 16(b). There are two concurrent factors contributing to voltage drops of both surfaces:

1. signal propagation along the transmission line (dependent on the time constant of the actuator);
2. the time constant is dynamically changing due to the instantaneous curvature of the actuator.

Separating these two processes is a rather complex procedure, however, based on Figure 16(b) we can argue that for the extreme cases of obstruction and free movement it is possible to visually detect different signals. Furthermore, the surface signals of the completely fixed sample describe a particular case where curvature is held constant. This means that the time constant remains unaltered, and, thus measured signals describe only the signal propagation along the CPC as a transmission line.

When actuator is completely fixed, the decay of voltage is roughly exponential on each surface. It is observed from the characteristics that the voltage along the electrodes is distributed unevenly and depends on the curvature of the freely bending actuator. The progress of appearance of a voltage drop along the compressed electrode (*V_R_*) is roughly similar to the previously fixed case. The irregular behavior of the voltage drop along the stretching electrode (*V_L_*) indicates that the resistance and apparent capacitance of the corresponding electrode are not constant. Although in Section 4 we showed that no element of capacitance is found in the surface electrode, it can be stated that this outcome complies with fore mentioned results where bending of a CPC actuator causes a significant change in apparent capacitance measured from the surface that is undergoing expansion (Section 3, Figure 7).

**Figure 17.**
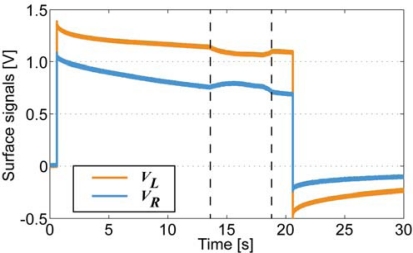
Voltage drops along the surface of the CPC actuator, free movement of the actuator was obstructed at the 14th second of the experiment.

Due to very distinctive alteration of both resistance and apparent capacitance, this type of system can be utilized as biomimetic vibrissae. Figure 17 shows voltage drops along each surface of a freely moving CPC actuator, however in approximately 14 seconds (*Δx* ≈ 5 mm) an opposing beam forces the actuator back to its initial state. This impediment is unmistakably visible in voltage drops of each surface, hence providing a self-sensing collision detecting CPC actuator. Interestingly this phenomenon appears to have symmetric nature on both voltage drop, thus indicating that this effect is not caused only by the alteration of surface resistance (that behaves differently on compression and expansion, Figure 8(a)) but instead, by a resultant effect of change in apparent capacitance.

## 6. Conclusions

Here we presented the determination of the sensorial properties of a carbon-polymer composite (CPC) actuator based on measurements conducted mainly on its surface electrode. It is shown that alteration in resistance of the electrode due to the bending is present for the CPC actuator as well as for the electrode itself. However, a noteworthy effect of capacitance is also measured from the surface electrode of the CPC laminate. When investigating the components of CPC individually, we demonstrate that no detectable capacitance is present on single surface electrode, hence, indicating to a purely resistive nature of CDC-based electrodes. Therefore, this apparent capacitance is caused by the coupling effect of the PVdF membrane separating two electrodes of the CPC actuator. Moreover, whenever measuring signals from any surface of a CPC actuator, one has to consider this capacitive coupling between electrodes that is easily detectable from the surface undergoing expansion. However, the apparent capacitance shows a good correlation to the bending curvature of a CPC sample, thus, providing another means to a position sensing with CPC.

As measurements of cross-electrode impedance indicate that no significant alteration of electrical parameters is detected on the separator membrane, most of the electrical phenomena observed during bending must, thus, be contributed to the properties of surface electrode.

Empirical resistive and capacitive effects are more prominent when measuring the surface that is being stretched as a result of actuation or external bending. A self-sensitivity of completely blocked or externally manipulated CPC actuator is also presented in this paper. This type of self-sensing system can be utilized as biomimetic vibrissae that allow tactile feedback.

Implementation of this type of tactile self-sensing CPC actuator in a robotic application is the subject of planned future work, along with a study into appropriate conditioning circuits to better benefit from the sensorial properties during an actuator’s work-cycle. Also, a study into how mechanical properties (such as elastic modulus) contribute to sensing and actuating properties of CPC is underway.
